# Clinical impact of the alpha-galactosidase A gene single nucleotide polymorphism -10C>T: A single-center observational study

**DOI:** 10.1097/MD.0000000000010669

**Published:** 2018-05-25

**Authors:** Daniel Oder, Dan Liu, Nurcan Üçeyler, Claudia Sommer, Kai Hu, Tim Salinger, Jonas Müntze, Bernhard Petritsch, Georg Ertl, Christoph Wanner, Peter Nordbeck, Frank Weidemann

**Affiliations:** a Department of Internal Medicine I, Division of Cardiology and Nephrology and Comprehensive Heart Failure Center Würzburg, University Hospital Würzburg; b Fabry Center for Interdisciplinary Therapy, University of Würzburg; c Department of Neurology, University Hospital Würzburg; d Department of Diagnostic and Interventional Radiology, University Hospital Würzburg, Würzburg; e Department of Medicine II, Katharinen-Hospital Unna, Unna, Germany.

**Keywords:** cardiomyopathy, Fabry disease, left ventricular hypertrophy, neuropathic pain, nonclassical phenotype, SNP

## Abstract

Single nucleotide polymorphisms (SNPs) in the alpha-galactosidase A gene region (GLA) have been discussed as potential cause of symptoms and organ manifestations similarly to those seen in Fabry disease (FD). However, due to scarce data, clinical implications remain limited. The aim of the present study was to investigate the clinical impact of -10C>T SNP in the GLA.

Prospective single-center observational study to determine the natural history and outcome of FD.

Subjects initially referred to the Fabry Center for Interdisciplinary Therapy Würzburg (FAZIT) for management of suspected FD (11 women, 2 men, mean age 42 ± 10 years) who were tested negative for coding *GLA* mutations but positive for the noncoding -10C>T SNP underwent comprehensive characterization for therapy recommendation.

All subjects reported at least 1 neurological, but no cardiac or renal symptoms. In 7 patients, pain of unknown etiology was reported and 3 patients had a history of cryptogenic stroke. In all patients, α-GAL activity was at a lower limit, ranging between 0.27 and 0.45 nmol/min per mg protein (reference: 0.4–1.0), while plasma Lyso-Gb3 levels remained normal (range 0.39 ± 0.33; reference: ≤0.9 ng/mL). For both hemizygous subjects investigated, brain magnetic resonance imaging revealed unspecific white matter lesions. One of these subjects had suffered from severe early-onset stroke, the other showed mild hypertrophic cardiomyopathy.

Presence of isolated heterozygous -10C >T SNP is not associated with clinically relevant symptoms or organ manifestations as seen in FD. Respective polymorphisms might, however, play a role in modifying disease severity in FD. Great care has to be taken in respective subjects suspected to suffer from nonclassical FD in order to prevent unnecessary Fabry-specific therapy.

**Strengths and limitations of this study**

- Comprehensive and detailed analyzation of diagnostic pitfalls and inadequate therapy planning in rare conditions.
- Highlighting potential modifying impact of genetic polymorphisms in Fabry disease.
- Small number of investigated subjects.
- Partly retrospective study design.
- Heterogeneity of study subjects.

## 1 Introduction

Fabry disease (FD) (OMIM 301500) is an X-linked lysosomal storage disorder taking origin from pathogenic mutations in the alpha-galactosidase A (α-GAL) gene region (*GLA*), ultimately leading to phenotypic characteristics and complications in detail reported before.^[1,2]^ Several modifying factors are discussed playing a key role in regard to phenotypic heterogeneity including age, gender, the underlying genotype, and whether therapy is accessible or not.^[3–11]^ Recent studies gave cumulative suspicion for respective modifying impact of certain single nucleotide polymorphisms (SNPs) in the noncoding *GLA* regions.^[12,13]^ However, due to scarce data and no guidelines available, clinical implications are problematic and therapy decisions have to be made on an individual basis. Variants in the coding regions directly affect the enzyme peptide sequence and, therefore, potentially also its structure and function. By contrast, the impact of changes in the noncoding enzyme introns is currently poorly understood and thus hardly predictable. At least one of these mutations, the intronic *GLA* mutation IVS4+919A>G has been proven to directly affect α-GAL composition by alternative splicing.^[12,14,15]^

The aim of the present study was to investigate the clinical impact of the *GLA* SNP -10C>T in patients referred with clinical suspicion of FD. One prominent focus was set on comprehensive demonstration of diagnostic pitfalls, which in part have led to prejudged initiation of Fabry-specific enzyme replacement therapy (ERT). This might be of special impact due to the fact that, aside from well-established ERT, several novel oral therapies are evolving.^[16,17]^

## 2 Methods

### 2.1 Study population

Since its establishment in the year 2001, more than 270 patients with genetically proven FD attended the Fabry Center for Interdisciplinary Therapy Würzburg (FAZIT) for specialized clinical evaluation and therapy planning. As reference center for the evaluation of FD, FAZIT is also approached for evaluation of subjects with clinical suspicion for FD who were initially tested negative for disease-causing *GLA* mutations. Fourteen of such subjects tested negative for coding *GLA* variants but positive for the *GLA* SNP -10C>T [rs2071225], IVS2–81–77-delCAGCC [rs5903184], IVS4-16A>G [rs2071397], and IVS6-22C>T [rs2071228] were referred for second opinion and therapy recommendations. In 2 of these subjects, ERT had already been initiated at a different site and was ongoing at the time of first presentation at our center. The group consisted of 2 men and 12 women with a mean age of 42 ± 10 years at their first visit. Study subjects had mainly been identified due to the presence of unspecific neurological manifestations, respectively, symptoms, indicative and associated with FD: 4 patients suffered from stroke of unknown origin at young age and had initially been identified during the Stroke In Young Fabry Patients (SIFAP) trial, 3 other patients were related to a clinically affected index patient and were identified by family screening,^[18,19]^ and 7 additional patients initially presented with neurological symptoms of unknown origin, such as persistent pain, vertigo, and/or hypo-/anhidrosis. Molecular analysis revealed that none of these patients had any genetic mutation typical for FD, but all had either 1 or a combination of several noncoding genetic SNP that have been recently described to be potentially associated with clinical appearance of FD.^[12]^ The 2 subjects already receiving ERT had both been enrolled in the SIFAP trial.^[18,19]^ From the 14 patients initially presenting with noncoding SNP, 1 female patient was excluded later from study evaluations, since a coding *GLA* mutation was detected in a second genetic analysis. This prospective single-center observational study was approved by the Ethics Committee of the University Hospital Würzburg. All patients provided written informed consent.

### 2.2 Genetic testing

All patients underwent a commercially available genetic analysis of the *GLA* region at the Molecular Genetics and Metabolism Laboratory, Munich, Germany and/or at Centogene AG, Rostock, Germany. After the DNA was extracted from peripheral blood samples, all coding regions as well as exon–intron splice junctions of the *GLA* gene region were analyzed using a targeted custom Next-Generation Sequencing platform.

### 2.3 Clinical evaluation

All subjects underwent comprehensive clinical examinations with focus on potential Fabry-associated organ involvement and symptoms.

### 2.4 Laboratory tests and biomarker analysis

Renal function was measured by 99-Technetium DTPA clearance or, if not available, by 24-h urine, urea- and creatinine-clearance; serum creatinine (crep2, COBAS, Roche Diagnostics, Mannheim, Germany, article number: 05168589 190); albuminuria and proteinuria were determined by urinary albumin-to-creatinine ratio and urinary protein-to-creatinine ratio. For detection of myocardial involvement, the N-terminal B-type natriuretic peptide (NT-proBNP; reference: <125 pg/mL; proBNP II, COBAS, Roche Diagnostics, article number: 04842464 190) and high-sensitive troponin T (hs-TnT; reference values: 0–14 pg/mL; Troponin T hs, COBAS, Roche Diagnostics, article number: 05092728 190) were measured as well-established cardiac biomarkers.^[20–23]^ Plasma Lyso-Gb3 and α-GAL enzyme activity were obtained from circulating blood samples and analyzed at Centogene as indicators for Fabry-specific disease activity.^[24–26]^ Plasma Lyso-Gb3 levels (reference: <0.9 ng/mL) were measured using high-performance liquid chromatography mass spectrometry.^[24]^ The α-GAL enzyme activity (reference: 0.4–1.0 nmol/min per mg protein) was quantified by fluorescence spectrometry.^[27]^

### 2.5 Echocardiography

Echocardiographic measurements were performed in accordance to the current recommendations for cardiac chamber quantification by echocardiography in adults from the American Society of Echocardiography and the European Association of Cardiovascular Imaging.^[28,29]^

The standard apical views of the left ventricle (LV) were acquired for off-line quantification of myocardial deformation values by 2-dimensional speckle tracking using ECHO-Pac Software (GE Vingmed Ultrasound AS, Horten, Norway) as reported before.^[30]^

### 2.6 Cardiac magnetic resonance imaging

Cardiac magnetic resonance imaging (MRI) was performed on a 3 Tesla full body scanner (MAGNETOM Trio, Siemens Healthcare, Erlangen, Germany) in breath-hold technique with electrocardiogram (ECG) triggering. After morphologic cine imaging using a steady-state free precession sequence, patients received Gadubutrol (Gadovist, Bayer Vital GmbH, Leverkusen, Germany) in a standard dose of 0.2 mmol/kg body weight per intravenous injection. Late gadolinium enhancement (LGE) images were acquired using T1-weighted inversion recovery imaging sequences (field of view 240 × 320 mm^2^, matrix size 165 × 256, echo time 1.2 ms, repetition time 3.0 ms, inversion time determined individually). The evaluation of LGE (presence vs absence) as marker for myocardial fibrosis^[31]^ was performed independently by 2 radiologists with extended experience in the field.

### 2.7 Brain MRI

All patients received a cranial MRI using a head coil on a 3 Tesla clinical MR scanner. Contiguous slices in axial and coronal orientation were acquired to cover the whole brain, including T1-, T2-, proton density-, and diffusion-weighted, as well as 2D/3D time-of-flight/fluid-attenuated inversion recovery (FLAIR) MR angiography pulse sequences. To semiquantitatively assess cerebral white matter lesion load, we applied the Fazekas score for each subject on the FLAIR sequences. The Fazekas score ranges from 0 to 3 with 0 = no or a single punctuate white matter lesion, 1 = multiple punctuate lesions, 2 = beginning confluence of lesions, and 3 = large confluent lesions.^[32]^

### 2.8 Quantitative sensory testing

Quantitative sensory testing (QST) was performed using a calibrated device (Somedic, Hörby, Sweden) and following a standardized procedure.^[33]^ All subjects were investigated at the right dorsal foot. Based on the log transformed raw values for each QST item a z-score sensory profile was calculated as follows: z-score = (value of the subject − mean value of controls)/standard deviation of controls. Negative z-scores indicate loss of sensation, while positive z-scores indicate gain of sensation. We determined cold and heat detection thresholds and the ability to sense temperature changes (thermal sensory limen) as a measure of small fiber function. Paradoxical heat sensation was recorded if subjects experienced cold as heat. We additionally determined the vibration detection threshold as a measure of large fiber function. Data were compared with published reference data.^[34]^

### 2.9 Electroneurography

Neurophysiological assessment of the right sural nerve (antidromic recording; surface electrodes) was done in all patients following standard procedures.^[35]^ Results were compared with our normal laboratory values: lower limit of normal for sural nerve sensory nerve action potential (SNAP) peak-to-peak amplitude 10 μV for patients <65 years of age, 5 μV for >65 years of age; sural nerve conduction velocity (NCV) 40 m/s for all adult ages.

### 2.10 Skin biopsy

Two skin punch biopsies (5-mm in diameter; device by Stiefel GmbH, Offenbach, Germany) were taken under local anesthesia as previously described,^[36]^ 1 from the distal lateral calf 10 cm above the malleolus and 1 from the back (at th12 level). Skin samples were fixed in 4% paraformaldehyde at 4°C for 2 h. Intraepidermal nerve fibers were visualized on 50-μm cryosections immunoreacted with antibodies against the pan-axonal marker protein–gene product 9.5 (PGP9.5; Ultraclone, Cambridge, UK, 1:800) with goat antirabbit IgG labeled with cyanine 3.18 fluorescent probe (Amersham, Buckinghamshire, UK 1:100; Cy3). Intraepidermal nerve fiber density was determined following published criteria.^[37]^

### 2.11 Statistical analysis

Continuous data are presented as absolute numbers, mean value ± standard deviation (MW ± SD), or as median value (quartiles). Categorical variables are presented as percentages. Differences on strain rate and strain values among basal, mid, and apical levels were compared using 1-way analysis of variance followed by Tukey multiple comparison post hoc tests as appropriate. Statistical significance is indicated by *P* value < .05. These data were analyzed using IBM SPSS Statistics, version 22.0 (SPSS Inc, Chicago, IL).

## 3 Results

### 3.1 Genetic testing

None of the subjects showed any coding *GLA* mutation in second evaluation, but all a variation at -10C>T *GLA* SNP. Ten subjects were heterozygous including at least one of the additional intronic variants IVS2-81-77delCAGCC, IVS4-16A>G, and IVS6-22C>T. Only 1 subject did not show any additional intronic variant. Two subjects were hemizygous and 1 homozygous for -10C>T, all of these having additional intronic *GLA* variants. The homozygous female subject initially also included in the study stood out with particularly severe clinical manifestations including cardiomyopathy and beginning kidney failure. This subject was tested positive for the heterozygous *GLA* mutation c.1196G>C p.W399S, which is known to be associated with classical Fabry phenotype, in a second genetic analysis.^[38]^ This subject was, therefore, excluded from further analysis. An overview of the results from genetic testing is given in Table 1.

**Table 1:**
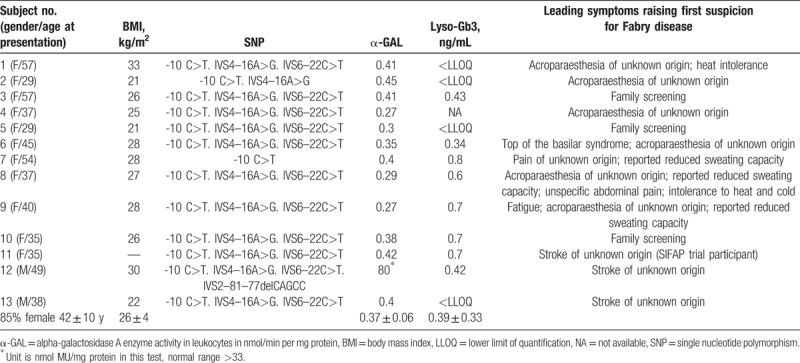
Patients demographics (n = 13).

### 3.2 Baseline characteristics

The study population (n = 13) was mainly female (85%), middle aged (42 ± 10 years), and had an average body mass index of 26 ± 4 kg/m^2^. Family screening was unremarkable with no noticeable history of typically Fabry-associated complications such as ventricular rhythm disturbances, sudden cardiac death, hypertrophic cardiomyopathy of unknown origin, chronic kidney disease, neuropathic pain, or stroke. Only in subject no. 8, 1 further family member reported suffering from more intense diarrhea, which was attributed to a known lactose intolerance but might also be associated with autonomic nervous system involvement frequently seen in FD. Laboratory analysis revealed an average α-GAL activity of 0.37 ± 0.06 nmol/min per mg protein and Lyso-Gb3 biomarker levels of 0.39 ± 0.33 ng/mL.

### 3.3 Symptoms raising suspicion for FD

Nine subjects (69.2%) initially reported pain in the extremities, 6 (46.2%) pain upon exercise, 4 (30.8%) a worsening of pain when exposed to heat or during exercise, and 3 (23.1%) reported either chronic pain or occurrence of acute pain attacks. Four subjects (30.8%) had a cerebrovascular insult in their medical history (no. 6 and 11–13). Detailed information can be found in Table 1.

### 3.4 Cardiac organ involvement

#### 3.4.1 Electrocardiogram

All subjects (100%) were in sinus rhythm, with resting blood pressure (126 ± 18/78 ± 12 mm Hg) and heart rate (71 ± 9 bpm) in normal ranges. None showed an atrioventricular or bundle branch block. One subject (no. 13) presented a positive Sokolow-Lyon Index with SV1+RV5 being 4.7 mV, and patient no. 12 showed T-wave inversions in I, aVL, and V6 in standard 12-lead ECG. Ergometry and 24-h holter ECG showed no pathologies suggesting ischemia or rhythm disorders in any patient.

#### 3.4.2 Cardiac biomarkers

NT-proBNP was 63 ± 44 pg/mL (reference: <125 pg/mL). hs-TnT^[39]^ was below the lower limit of quantification in 10 out of 13 subjects (<5 pg/mL, reference: 0–14 pg/mL), and in normal ranges in the remaining 3 subjects (no. 1: 7.9 pg/mL; no. 7: 8.3 pg/mL; and no. 12: 9.6 pg/mL; Table 2).

**Table 2:**
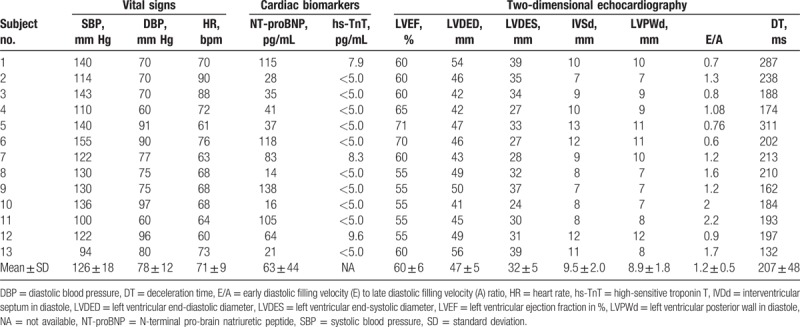
Cardiac characteristics in subjects with -10 C>T single nucleotide polymorphisms (n = 13).

#### 3.4.3 Cardiac imaging modalities

**Figure 1:**
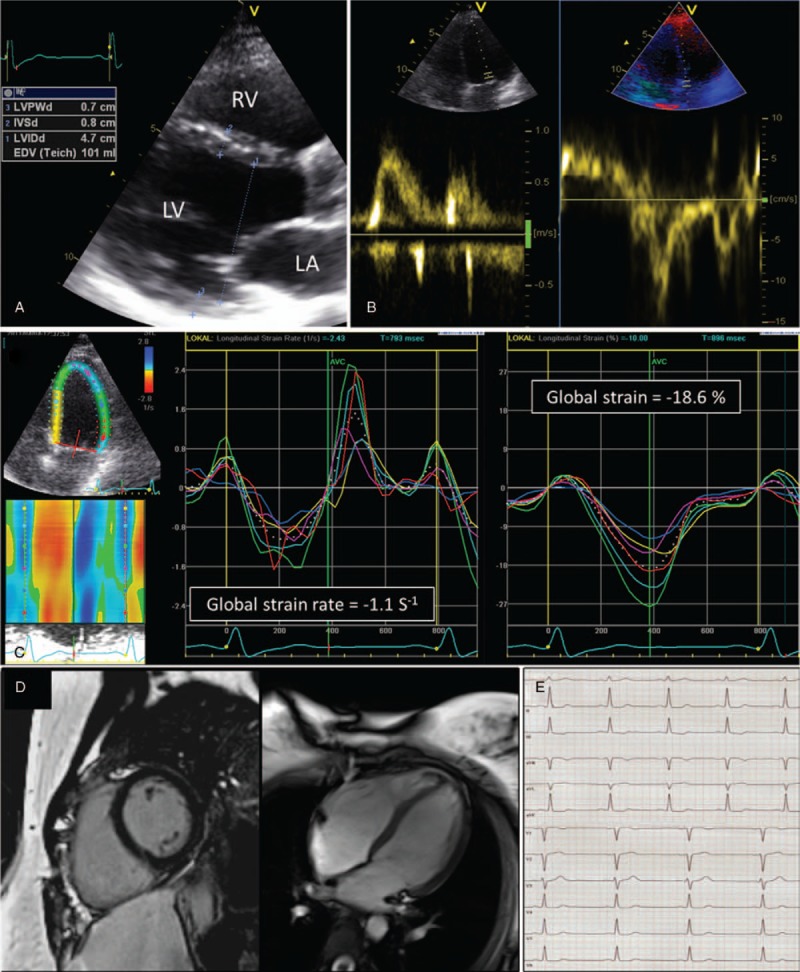
Representative example of 2-dimensional echocardiography (A and B), speckle tracking imaging (C), cardiac magnetic resonance imaging (D), and resting electrocardiogram (E) in a patient with reported heterozygous -10 C>T single nucleotide polymorphisms (patient no. 8). None of the cardiac imaging modalities revealed any cardiac pathology. Cardiac morphology and function remained normal. Furthermore, speckle tracking investigations demonstrated normal global and regional myocardial deformation values, negative for myocardial fibrosis as seen in Fabry cardiomyopathy.

**Figure 2:**
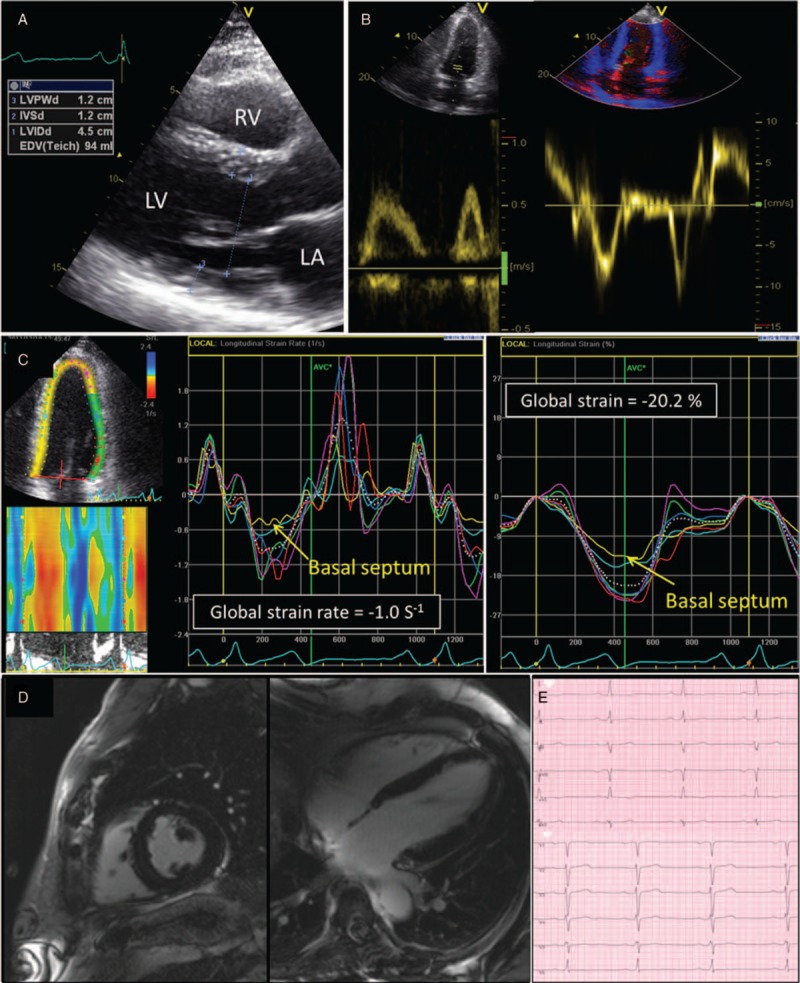
Representative example of 2-dimensional echocardiography (A and B), speckle tracking imaging (C), cardiac MRI (D), and resting electrocardiogram (E) in a patient with reported hemizygous -10 C>T single nucleotide polymorphisms (patient no. 12). Echocardiography and cardiac MRI revealed mild left ventricular hypertrophy (interventricular septal wall thickness is 12 mm). Although global systolic function and diastolic function remain normal, a significantly reduced strain rate and strain at the septal basal segment is evidenced by speckle tracking hinting on a myocardial pathology. MRI = magnetic resonance imaging.

**Figure 3:**
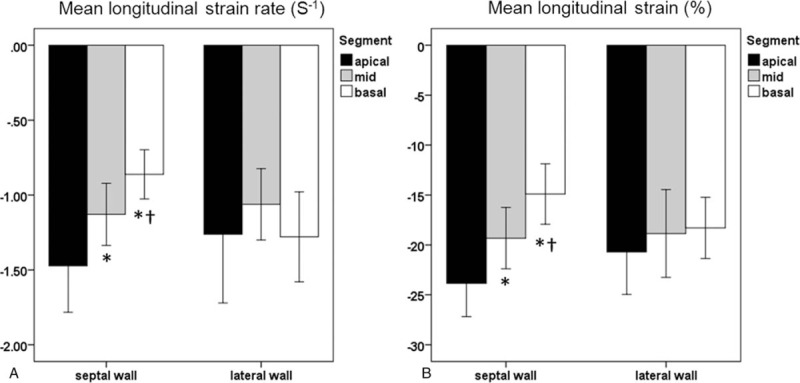
Speckle tracking derived longitudinal systolic strain rate (A) and strain (B) in the sepal and lateral walls. The bar plot demonstrates base-to-apex longitudinal systolic strain rate/strain gradient in the septum, presenting normal values at the apical segment and gradually reduced values at the middle and basal segments.

The interventricular septum and the posterior wall thickness were 9.5 ± 2.0 and 8.9 ± 1.8 mm, whereas LV end-systolic and end-diastolic diameters revealed 32 ± 5 and 47 ± 5 mm, respectively. E/A ratio was 1.2 ± 0.5 and the deceleration time 207 ± 48 ms in standard 2-dimensional echocardiography (Table 2). All subjects received a cardiac MRI, except for 1 subject who refused to undergo this investigation. Cardiac MRI revealed no apparent abnormality in any heterozygous patient (Fig. 1), but a tendency for cardiac hypertrophy in 1 hemizygous patient (Fig. 2). The normalized cardiac muscle mass (69 ± 23 g/m^2^), the normalized end-systolic and end-diastolic volumes (25 ± 6 and 68 ± 7 mL/m^2^), the normalized stroke volume (44 ± 7 mL/m^2^), the LVEF (64 ± 7%), and the cardiac index (3.3 ± 0.9 L/min per m^2^) were all in the normal range (Table 3). None of the subjects showed detectable LGE in cardiac MRI. The global strain rate from echocardiography was −1.04 ± 0.14 S^−1^, and global strain −18.9 ± 1.7% (Fig. 3).

**Table 3:**
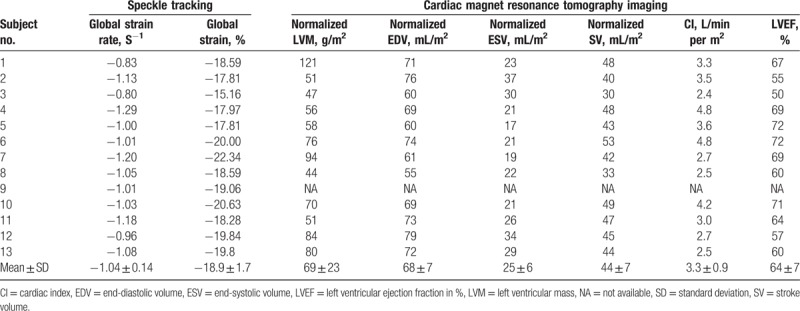
Two-dimensional speckle tracking and cardiac magnetic resonance imaging in subjects with -10 C>T single nucleotide polymorphisms (n = 13).

### 3.5 Renal organ involvement

The mean glomerular filtration rate based on urinary samples was measured as 88.3 ± 17.2 mL/min, and as 120.8 ± 22.6 mL/min in 99-Technetium DTPA-Clearance. The estimated glomerular filtration rate was calculated as 96.1 ± 24.9 mL/min per 1.73 m^2^. Mean serum creatinine was 0.8 ± 0.2 mg/dL. Mild proteinuria was found in 4 (28.6%), and albuminuria in 2 (14.3%) subjects’ urinary samples (Table 4). None of the patients was in need of a renal replacement therapy including hemodialysis nor kidney transplantation.

**Table 4:**
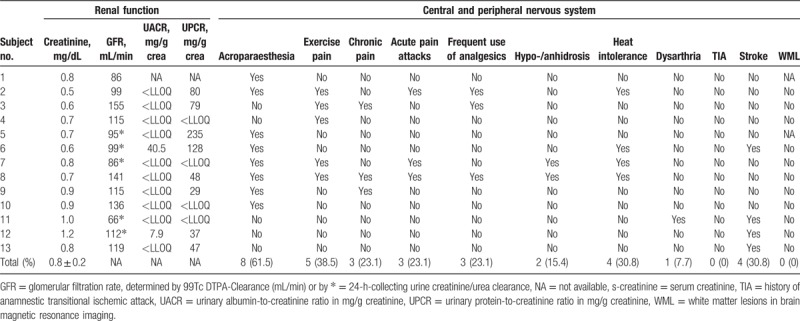
Renal function and anamnestic neurologic impairments in patients with −10 C>T single nucleotide polymorphisms before extensive neurological investigation was performed.

### 3.6 Neurological organ involvement

Twelve subjects underwent an extensive and specialized neurological assessment. Of these, all but 1 patient showed a normal neurological basic examination. One patient had residual symptoms from a left hemispheric cerebral stroke in terms of aphasia and spastic ride-sided hemiparesis.

#### 3.6.1 Pain

None of the subjects reported any typical Fabry-associated pain attacks, pain crises, evoked pain, or permanent pain.^[40]^ Five of 12 subjects reported no pain at all (Table 4). Of those who reported pain, the following underlying pathologies were found in 1 patient each: severe joint pain associated with arthrosis; pain due to carpal tunnel syndrome; chronic tension type headache and migraine; pain in small hand joints and shoulders associated with HLA-B27 positive rheumatoid arthritis. In 3 subjects, the reported pain could not be related to an underlying disease: 1 subject described irregular and episodic pain in arms, hands, and joints twice a week, 1 had pain in the knee joints after physical activity, and 1 described episodic nocturnal pain in her hands.

#### 3.6.2 Nerve conduction studies

Nerve conduction studies of the right sural nerve were normal in all cases but 1 (median SNAP amplitude 22 μV, range 12–51 μV; median NCV 52 m/s, range 43–64 m/s). One woman had a reduced SNAP amplitude of 3.1 μV, while NCV was in the normal range.

#### 3.6.3 Quantitative sensory testing

Compared with the normative values published before,^[34]^ thermal and pain perception and pain thresholds measured at the dorsal foot were normal in all cases.

#### 3.6.4 Skin biopsies

Skin punch biopsies were obtained from the lateral lower leg and the back in 7 patients. Median intraepidermal nerve fiber density was measured and found normal in all cases but one (lower leg: 11 fibers/mm, range 9–20 fibers/mm; back: 22 fibers/mm, range 20–54 fibers/mm). In 1 male subject (no. 13), intraepidermal nerve fiber density at the lower leg was slightly reduced to 4.4 fibers/mm (normal range 6–12 fibers/mm).

#### 3.6.5 Stroke

Four subjects (2 men and 2 women) had a history of a previous cerebral stroke: 1 had suffered from embolic strokes in the right median cerebral artery territory, 1 in the posterior cerebral artery area territory without residual symptoms, 1 had a recanalized distal internal carotid artery occlusion with residual aphasia and spastic ride-sided hemiparesis, and 1 an infarction of the rostral brainstem and cerebral hemispheral regions due to cryptogenic embolism in the distal basilar artery. Events suspicious for transitory ischemic attacks or hearing loss^[41]^ were not reported by any subject.

#### 3.6.6 Brain MRI

**Figure 4:**
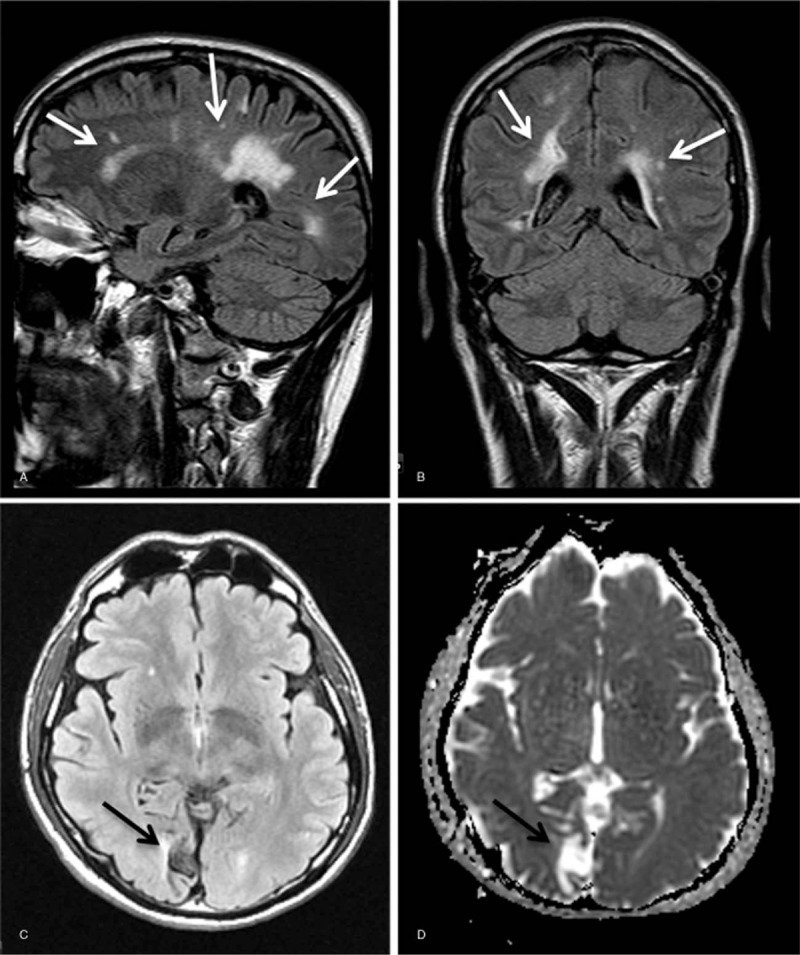
Representative examples of performed brain MRI investigations for evaluation of brain tissue involvement in patients with hemizygous -10 C>T single nucleotide polymorphisms. Brain MRI reveals micro- and/or macrovasculopathy (A and B, patient no. 12, white arrows) and/or ischemic encephalopathy (C and D, patient no. 13, black arrows). MRI = magnetic resonance imaging.

Brain MRI scans were available for 11 subjects. One female subject had confluent white matter lesions that had initially led to the diagnosis of multiple sclerosis in her medical history. One male subject had elongated vertebrobasilar cerebral arteries and leukoencephalopathy. This subject had had embolic strokes in the right median cerebral artery territory without residual symptoms as described above. In 1 male subject, brain MRI displayed cerebral ischemia in the right posterior cerebral artery area without white matter lesions. One female subject showed an ischemic stroke in the median cerebral artery area. This subject suffered from residual aphasia and spastic hemiparesis, as described above. Figure 4 shows brain MRI from subjects 12 and 13.

In all other cases, normal MRI scans were found with only minor microangiopathic changes. The Fazekas score was 0 in 3 cases, 1 in 5 cases, 2 in 1 case, and 3 in 1 case.

### 3.7 Nonspecific Fabry-related symptoms

#### 3.7.1 Sweating capacities

According to the subjects’ reports, anhidrosis was not present in any case, while 2 subjects reported reduced sweating capacities (hypohidrosis). Neither subject reported hyperhidrosis. However, sweating capacities were only obtained through anamnesis, but were not quantified.

#### 3.7.2 Gastrointestinal symptoms

Five study subjects reported unspecific gastrointestinal disorders. In detail, 3 claimed occasional diarrhea and flatulence after specific meals (patient no. 2, 4, and 6); 2 reported irregular obstipation accompanied by mild abdominal cramping and pain (patients no. 8 and 9).

## 4 Discussion

An extensive and comprehensive clinical, biochemical, and imaging study was performed in subjects with well-described noncoding *GLA* SNP suspected to be causal for the development of symptoms as seen in mild variants of nonclassical FD.^[12,13]^ Our data show that all subjects primarily suffered from neurological symptoms, which led to initial suspicion for FD triggering further investigations and in some cases even triggered Fabry-specific therapy at different sites before second opinion was obtained at FAZIT.

### 4.1 Neurological involvement in -10 C>T SNP

Patients with FD have a high incidence not only for neurological disorders and symptoms such as Fabry-associated pain, but also a particularly high risk for cerebrovascular events such as young-aged cryptogenic stroke.^[18,19]^ The SIFAP study investigated the prevalence of FD in a large cohort of respective patients with stroke of unknown origin at specifically young age.^[18]^ Many of these investigated study participants presented neurological symptoms as often seen in FD, and therefore were subsequently clinically misinterpreted and diagnosed as Fabry patients, even in cases where no disease causing mutation could be found. It was hypothesized that there might be further genetic modifiers besides classical mutations within the *GLA* region, which might lead to pathological conditions of clinically relevant FD.^[18]^ Advanced understanding of FD pathophysiology, the availability of new prognostic biomarkers, and modern imaging techniques allow the re-evaluation of earlier diagnoses, which is crucial particularly with regard to the indication and potential benefit of Fabry-specific therapy. In line with previous findings, we found that all subjects in the present study reported at least 1, and 28.6% three or more neurological symptoms of otherwise unexplained origin. Four of these subjects (30.8%) also suffered from an ischemic cerebral event at young age (31, 36, 44, and 49 years old). In addition, 1 subject was initially sent with the diagnosis of a pronounced (vascular) encephalopathy. This relatively high incidence of neurological symptoms is in line with the suggestions from recent studies on noncoding *GLA* SNP, which mainly focused on neurological symptoms. However, thorough neurological examination revealed physiological results in all cases except for 1 subject with residual symptoms and signs after cerebral stroke.

### 4.2 Heterozygous SNP

Eleven subjects in the present study were heterozygous and neither of them had clinical signs of a beginning or manifest cardiomyopathy nor renal organ involvement. Cardiac MRI, speckle tracking strain, and strain rate did not reveal signs of cardiac fibrosis, pathologic muscle stiffness, or rigidity. Furthermore, NT-proBNP and hs-TnT were all within normal range, indicating that no cardiac involvement analogous to the Fabry cardiomyopathy is triggered by noncoding *GLA* SNP. Regarding renal involvement, all measured parameters were in normal range with good glomerular filtrations rates in all heterozygous subjects. Only 2 subjects showed mild proteinuria and 1 patient had albuminuria. Similarly, despite initially raised suspicions, neurological assessment did not reveal typical FD-related findings. Thus, the main result of our small case study is that patients with heterozygous *GLA* SNP do not develop clinically relevant signs or symptoms as typically seen in neither classical nor nonclassical Fabry phenotypes. Thus, great care should be taken into account when discussing therapeutic options which in respective cases had led to incorrect ERT initiation at different sites before a second opinion was gained at FAZIT. This also highlights the need of specialized centers of reference in both diagnosis and therapy planning of FD.

### 4.3 Hemi/homozygous SNP

While in the present study all subjects with heterozygous *GLA* SNP did not suffer from any organ manifestations or typical symptoms as usually seen in FD, subjects with hemi-/homozygous genetic SNP appeared to show some clinical symptoms and to some extend also pathological organ manifestations.

Three initially screened subjects were hemi-/homozygous, with one of these also diagnosed with an additional rare mutation eventually causing classical FD; this patient suffered from a typical Fabry cardiomyopathy and nephropathy and was excluded from the final study analysis due to the detection of an additional coding *GLA* mutation. One hemizygous subject (no. 12) suffered from a severe subcortical encephalopathy, repetitive strokes (n ≥ 3) with the first occurring at the age of 47, showed an early cardiomyopathy including mild myocardial fibrosis, microproteinuria, and a reduced intraepidermal nerve fiber density in distal skin. These findings suggest that hemizygous noncoding *GLA* SNP might indeed show clinical symptoms and organ involvement as seen in classical FD. On the other hand, the second hemizygous subject (no. 13) presented with normal cardiac and renal morphology and function. However, this subject was still relatively young and also showed manifest cerebral involvement, with stroke in the posterior cerebral artery territory without residual symptoms occurring at the age of only 36. Patient no. 14 stands out with particularly severe renal and cardiac dysfunction. However, this is well-explained by the existence of the later found coding *GLA* mutation.

Taken together, our data suggest that subjects with hemi-/homozygous *GLA* SNP might suffer from symptoms as seen in a mild or late onset variant of FD. This might explain, why 1 of 3 patients in this group showed severe neurological involvement including stroke, but—at the age of 42 today—still no detectable cardiac or renal organ involvement or dysfunction, which might, however, manifest later on in life. To further clarify the impact of homozygosity in subjects with SNP in the *GLA* gene it seems reasonable to complete a comprehensive diagnostic protocol including clinical, biochemical, and imaging data in these subjects in the future, eventually also including biopsy of the skin, kidneys, and the heart.

### 4.4 Clinical impact and diagnostic pitfalls in FD

On the basis of the current findings, subjects with the investigated noncoding *GLA* SNP might—in case of homozygosity—suffer from symptoms as seen in a mild variant of FD. In addition, until then unknown further genetic modifiers might take additional clinical impact. Thus, in cases when clinical status strongly suggests FD despite contradictory negative results of genetic testing, re-evaluation of the molecular analysis is mandatory. The question, whether homozygous *GLA* SNP might play a role as modifying factors eventually leading to somehow clinically relevant disease manifestations cannot be answered due to these study limitations. In case of proven organ involvement, it has to be considered individually how these organ manifestations can be adequately treated and quality of life preserved or increased. Since the present study gave also suspicion of manifest organ involvement, the final therapeutic regime in patients with hemi-/homozygous noncoding *GLA* SNP naturally should be made in an individual case-by-case decision. However, as none of the investigated subjects in the present study showed the typical clinical aggregate of symptoms and organ manifestations as typically seen in patients with classical FD, Fabry-specific therapy does not seem reasonable. Respective subjects might, however, benefit from follow-up evaluation and adjunctive therapy concepts including angiotensin II receptor blockers/angiotensin-converting-enzyme inhibitors in case of renal impairments. Our investigations also highlight the burden of diagnostic pitfalls in rare hereditary pathologies as metabolic disorders and the importance of reference centers of excellence not only in diagnosing but also excluding respective rare conditions.

## 5 Conclusions

The noncoding *GLA* -10C>T SNP does not lead to clinically relevant symptoms or organ manifestations as seen in classical or late onset FD. However, the study results indicate that patients with hemi-/homozygous SNP might show at least some clinically relevant symptoms and also manifest organ involvement comparable to patients with FD, which might indicate a modifying impact of respective polymorphisms. This might be of special interest, especially regarding the question of intrafamily severity differences in FD. Due to the small number of hemizygous study subjects, the clinical and modifying impact of hemi-/homozygosity should therefore be investigated further in a larger patient study with proven FD.

## Acknowledgments

The authors thank Mrs Irina Turkin for her dedicated long-standing work in the Fabry Center Würzburg.

## Author contributions

**Substantial contributions to the conception and design of the work:** Daniel Oder, Christoph Wanner, Peter Nordbeck, Frank Weidemann. Acquisition, analysis, and/or interpretation of data: all authors. Drafting the work or revising it critically for important intellectual content: all authors. Final approval of the version to be published: all authors. Agreement to be accountable for all aspects of the work in ensuring that questions related to the accuracy or integrity of any part of the work are appropriately investigated and resolved: all authors.

**Conceptualization:** Daniel Oder, Nurcan Üçeyler, Claudia Sommer, Christoph Wanner, Peter Nordbeck, Frank Weidemann.

**Data curation:** Daniel Oder, Dan Liu, Nurcan Üçeyler, Claudia Sommer, Kai Hu, Tim Salinger, Jonas Müntze, Bernhard Petritsch, Georg Ertl, Christoph Wanner, Peter Nordbeck, Frank Weidemann.

**Formal analysis:** Daniel Oder, Dan Liu, Nurcan Üçeyler, Claudia Sommer, Kai Hu, Bernhard Petritsch, Christoph Wanner, Peter Nordbeck, Frank Weidemann.

**Funding acquisition:** Georg Ertl, Christoph Wanner, Peter Nordbeck, Frank Weidemann.

**Investigation:** Daniel Oder, Dan Liu, Nurcan Üçeyler, Claudia Sommer, Kai Hu, Tim Salinger, Jonas Müntze, Bernhard Petritsch, Christoph Wanner, Peter Nordbeck, Frank Weidemann.

**Methodology:** Daniel Oder, Dan Liu, Nurcan Üçeyler, Claudia Sommer, Christoph Wanner, Peter Nordbeck, Frank Weidemann.

**Project administration:** Daniel Oder, Claudia Sommer, Christoph Wanner, Peter Nordbeck, Frank Weidemann.

**Resources:** Christoph Wanner, Peter Nordbeck, Frank Weidemann.

**Software:** Dan Liu, Bernhard Petritsch, Peter Nordbeck.

**Supervision:** Nurcan Üçeyler, Claudia Sommer, Georg Ertl, Christoph Wanner, Peter Nordbeck, Frank Weidemann.

**Validation:** Daniel Oder, Dan Liu, Nurcan Üçeyler, Claudia Sommer, Bernhard Petritsch, Peter Nordbeck, Frank Weidemann.

**Visualization:** Daniel Oder, Dan Liu, Claudia Sommer, Peter Nordbeck.

**Writing – original draft:** Daniel Oder, Peter Nordbeck.

**Writing – review and editing:** Daniel Oder, Dan Liu, Nurcan Üçeyler, Claudia Sommer, Kai Hu, Tim Salinger, Jonas Müntze, Bernhard Petritsch, Georg Ertl, Christoph Wanner, Peter Nordbeck, Frank Weidemann.

## References

[1] Desnick RJ Brady R Barranger J Fabry disease, an under-recognized multisystemic disorder: expert recommendations for diagnosis, management, and enzyme replacement therapy. Ann Intern Med 2003;138:338–46. 12585833 10.7326/0003-4819-138-4-200302180-00014

[2] Mehta A Ricci R Widmer U Fabry disease defined: baseline clinical manifestations of 366 patients in the Fabry Outcome Survey. Eur J Clin Invest 2004;34:236–42. 15025684 10.1111/j.1365-2362.2004.01309.x

[3] MacDermot KD Holmes A Miners AH Anderson-Fabry disease: clinical manifestations and impact of disease in a cohort of 60 obligate carrier females. J Med Genet 2001;38:769–75. 11732485 10.1136/jmg.38.11.769 PMC1734754

[4] MacDermot KD Holmes A Miners AH Anderson-Fabry disease: clinical manifestations and impact of disease in a cohort of 98 hemizygous males. J Med Genet 2001;38:750–60. 11694547 10.1136/jmg.38.11.750 PMC1734761

[5] Lukas J Giese AK Markoff A Functional characterisation of alpha-galactosidase a mutations as a basis for a new classification system in Fabry disease. PLoS Genet 2013;9:e1003632. 23935525 10.1371/journal.pgen.1003632 PMC3731228

[6] Rombach SM Smid BE Linthorst GE Natural course of Fabry disease and the effectiveness of enzyme replacement therapy: a systematic review and meta-analysis: effectiveness of ERT in different disease stages. J Inherit Metab Dis 2014;37:341–52. 24492980 10.1007/s10545-014-9677-8

[7] Oder D Uceyler N Liu D Organ manifestations and long-term outcome of Fabry disease in patients with the GLA haplotype D313Y. BMJ Open 2016;6:e010422. 10.1136/bmjopen-2015-010422 PMC4838741 27059467

[8] Oder D Liu D Hu K alpha-Galactosidase A genotype N215S induces a specific cardiac variant of Fabry disease. Circ Cardiovasc Genet 2017;10:e001691. 29018006 10.1161/CIRCGENETICS.116.001691

[9] Arends M Wanner C Hughes D Characterization of classical and nonclassical Fabry disease: a multicenter study. J Am Soc Nephrol 2017;28:1631–41. 27979989 10.1681/ASN.2016090964 PMC5407735

[10] Arends M Biegstraaten M Hughes DA Retrospective study of long-term outcomes of enzyme replacement therapy in Fabry disease: analysis of prognostic factors. PLoS ONE 2017;12:e0182379. 28763515 10.1371/journal.pone.0182379 PMC5538714

[11] Weidemann F Maier SK Stork S Usefulness of an implantable loop recorder to detect clinically relevant arrhythmias in patients with advanced Fabry cardiomyopathy. Am J Cardiol 2016;118:264–74. 27265676 10.1016/j.amjcard.2016.04.033

[12] Schelleckes M Lenders M Guske K Cryptogenic stroke and small fiber neuropathy of unknown etiology in patients with alpha-galactosidase A -10T genotype. Orphanet J Rare Dis 2014;9:178. 25423912 10.1186/s13023-014-0178-5 PMC4255940

[13] Tuttolomondo A Duro G Pecoraro R A family with various symptomatology suggestive of Anderson-Fabry disease and a genetic polymorphism of alpha galactosidase A gene. Clin Biochem 2015;48:55–62. 25281798 10.1016/j.clinbiochem.2014.09.018

[14] Ishii S Nakao S Minamikawa-Tachino R Alternative splicing in the alpha-galactosidase A gene: increased exon inclusion results in the Fabry cardiac phenotype. Am J Hum Genet 2002;70:994–1002. 11828341 10.1086/339431 PMC379133

[15] Liu HC Lin HY Yang CF Globotriaosylsphingosine (lyso-Gb3) might not be a reliable marker for monitoring the long-term therapeutic outcomes of enzyme replacement therapy for late-onset Fabry patients with the Chinese hotspot mutation (IVS4+919G>A). Orphanet J Rare Dis 2014;9:111. 25047006 10.1186/s13023-014-0111-y PMC4223723

[16] Guerard N Oder D Nordbeck P Lucerastat, an iminosugar for substrate reduction therapy: tolerability, pharmacodynamics, and pharmacokinetics in patients with Fabry disease on enzyme replacement. Clin Pharmacol Ther 2018;103:703–11. 28699267 10.1002/cpt.790

[17] Germain DP Hughes DA Nicholls K Treatment of Fabry's disease with the pharmacologic chaperone migalastat. N Engl J Med 2016;375:545–55. 27509102 10.1056/NEJMoa1510198

[18] Rolfs A Bottcher T Zschiesche M Prevalence of Fabry disease in patients with cryptogenic stroke: a prospective study. Lancet 2005;366:1794–6. 16298216 10.1016/S0140-6736(05)67635-0

[19] Rolfs A Fazekas F Grittner U Acute cerebrovascular disease in the young: the Stroke in Young Fabry Patients study. Stroke 2013;44:340–9. 23306324 10.1161/STROKEAHA.112.663708

[20] Coats CJ Parisi V Ramos M Role of serum N-terminal pro-brain natriuretic peptide measurement in diagnosis of cardiac involvement in patients with Anderson–Fabry disease. Am J Cardiol 2013;111:111–7. 23040658 10.1016/j.amjcard.2012.08.055

[21] Torralba-Cabeza MA Olivera S Hughes DA Cystatin C and NT-proBNP as prognostic biomarkers in Fabry disease. Mol Genet Metab 2011;104:301–7. 21795086 10.1016/j.ymgme.2011.06.021

[22] Feustel A Hahn A Schneider C Continuous cardiac troponin I release in Fabry disease. PLoS ONE 2014;9:e91757. 24626231 10.1371/journal.pone.0091757 PMC3953535

[23] Tanislav C Feustel A Franzen W Persistent increase in cardiac troponin I in Fabry disease: a case report. BMC Cardiovasc Disord 2011;11:6. 21281467 10.1186/1471-2261-11-6 PMC3039626

[24] Aerts JM Groener JE Kuiper S Elevated globotriaosylsphingosine is a hallmark of Fabry disease. Proc Natl Acad Sci USA 2008;105:2812–7. 18287059 10.1073/pnas.0712309105 PMC2268542

[25] Togawa T Kodama T Suzuki T Plasma globotriaosylsphingosine as a biomarker of Fabry disease. Mol Genet Metab 2010;100:257–61. 20409739 10.1016/j.ymgme.2010.03.020

[26] Niemann M Rolfs A Stork S Gene mutations versus clinically relevant phenotypes: lyso-Gb3 defines Fabry disease. Circ Cardiovasc Genet 2014;7:8–16. 24395922 10.1161/CIRCGENETICS.113.000249

[27] Caudron E Prognon P Germain DP Enzymatic diagnosis of Fabry disease using a fluorometric assay on dried blood spots: an alternative methodology. Eur J Med Genet 2015;58:681–4. 26520229 10.1016/j.ejmg.2015.10.014

[28] Lang RM Badano LP Mor-Avi V Recommendations for cardiac chamber quantification by echocardiography in adults: an update from the American Society of Echocardiography and the European Association of Cardiovascular Imaging. Eur Heart J Cardiovasc Imaging 2015;16:233–70. 25712077 10.1093/ehjci/jev014

[29] Lang RM Badano LP Mor-Avi V Recommendations for cardiac chamber quantification by echocardiography in adults: an update from the American Society of Echocardiography and the European Association of Cardiovascular Imaging. J Am Soc Echocardiogr 2015;28:1–39. 25559473 10.1016/j.echo.2014.10.003

[30] Kramer J Niemann M Liu D Two-dimensional speckle tracking as a non-invasive tool for identification of myocardial fibrosis in Fabry disease. Eur Heart J 2013;34:1587–96. 23520186 10.1093/eurheartj/eht098

[31] Beer M Weidemann F Breunig F Impact of enzyme replacement therapy on cardiac morphology and function and late enhancement in Fabry's cardiomyopathy. Am J Cardiol 2006;97:1515–8. 16679096 10.1016/j.amjcard.2005.11.087

[32] Fazekas F Chawluk JB Alavi A MR signal abnormalities at 1.5 T in Alzheimer's dementia and normal aging. AJR Am J Roentgenol 1987;149:351–6. 3496763 10.2214/ajr.149.2.351

[33] Rolke R Baron R Maier C Quantitative sensory testing in the German Research Network on Neuropathic Pain (DFNS): standardized protocol and reference values. Pain 2006;123:231–43. 16697110 10.1016/j.pain.2006.01.041

[34] Magerl W Krumova EK Baron R Reference data for quantitative sensory testing (QST): refined stratification for age and a novel method for statistical comparison of group data. Pain 2010;151:598–605. 20965658 10.1016/j.pain.2010.07.026

[35] Kimura J Electrodiagnosis in Diseases of Nerve and Muscle: Principles and Practice. New York, NY: Oxford University Press; 2001.

[36] Uceyler N Kafke W Riediger N Elevated proinflammatory cytokine expression in affected skin in small fiber neuropathy. Neurology 2010;74:1806–13. 20513817 10.1212/WNL.0b013e3181e0f7b3

[37] Lauria G Cornblath DR Johansson O EFNS guidelines on the use of skin biopsy in the diagnosis of peripheral neuropathy. Eur J Neurol 2005;12:747–58. 16190912 10.1111/j.1468-1331.2005.01260.x

[38] Eng CM Ashley GA Burgert TS Fabry disease: thirty-five mutations in the alpha-galactosidase A gene in patients with classic and variant phenotypes. Mol Med 1997;3:174–82. 9100224 PMC2230047

[39] Seydelmann N Liu D Kramer J High-sensitivity troponin: a clinical blood biomarker for staging cardiomyopathy in Fabry disease. J Am Heart Assoc 2016;5:e002114. 27247331 10.1161/JAHA.115.002839 PMC4937248

[40] Uceyler N Ganendiran S Kramer D Characterization of pain in Fabry disease. Clin J Pain 2014;30:915–20. 24121530 10.1097/AJP.0000000000000041

[41] Koping M Shehata-Dieler W Cebulla M Cardiac and renal dysfunction is associated with progressive hearing loss in patients with Fabry disease. PLoS ONE 2017;12:e0188103. 29161295 10.1371/journal.pone.0188103 PMC5697846

